# Remnant cholesterol–inflammation index and depressive symptoms in middle-aged and older adults with study-defined low bone mass: component-specific analysis and supportive clinical corroboration

**DOI:** 10.3389/fpubh.2026.1976857

**Published:** 2026-10-09

**Authors:** Bin Deng, Wei Zhang, Xiaoduo Zhang, Zhijian Peng, Wenhua Liu

**Affiliations:** 1 The Seventh Clinical Medical School of Guangzhou University of Chinese Medicine, Shenzhen Bao'an Chinese Medicine Hospital, Shenzhen, Guangdong, China; 2 Shenzhen Bao'an Authentic TCM Therapy Hospital, Shenzhen, Guangdong, China

**Keywords:** depressive symptoms, C-reactive protein, low bone mass, NHANES, middle-aged and older adults, remnant cholesterol, remnant cholesterol–inflammation index

## Abstract

**Background:**

Low bone mass and depressive symptoms commonly coexist in later life, but their shared metabolic–inflammatory profile is poorly characterized. We examined the remnant cholesterol–inflammation index (RCII) within adults aged ≥50 years with study-defined low bone mass, evaluated its component-specific associations, and assessed directional consistency in a separate outcome-enriched clinical sample.

**Methods:**

This cross-sectional analysis used survey-weighted NHANES data. Logistic and linear regression models examined depressive-symptom screening positivity and PHQ-9 scores, respectively. Restricted cubic splines were used to evaluate dose–response associations, and component-specific models jointly included standardized log-transformed calculated remnant cholesterol (RC) and cycle-specific C-reactive protein (CRP). The primary association was examined in an outcome-enriched clinical sample using the fixed NHANES standard-deviation (SD) scale.

**Results:**

Among 1,743 NHANES participants, 139 screened positive (PHQ-9 ≥10). Each survey-weighted SD increment in ln(RCII) was associated with screening positivity (OR, 1.52; 95% CI, 1.15–2.00; *P* = 0.004). Odds were higher in the highest versus lowest weighted quartile (OR, 3.00; 95% CI, 1.40–6.43; *P* for trend = 0.002), with no strong evidence of nonlinearity (*P* = 0.874). Higher ln(RCII) was also associated with higher PHQ-9 scores (β per SD, 0.38; 95% CI, 0.13–0.63; *P* = 0.004). In the mutually adjusted component model, the cycle-specific CRP measurement remained associated with screening positivity (OR per SD, 1.44; 95% CI, 1.12–1.84; *P* = 0.005), whereas calculated RC did not (OR, 1.14; 95% CI, 0.89–1.47; *P* = 0.284); the direct standardized coefficient contrast was imprecise and nonsignificant (*P* = 0.179). The direction of association was consistent in the outcome-enriched clinical sample (*N* = 150; 50 events; adjusted OR per fixed NHANES SD, 3.29; 95% CI, 1.92–5.64; *P* < 0.001).

**Conclusion:**

Higher RCII was associated with greater depressive-symptom burden among adults with low bone mass, with supportive directional clinical corroboration. Prospective studies with clinically confirmed outcomes are needed.

## 1 Introduction

Osteoporosis and low bone mass rank among the most prevalent disorders of aging. Globally, osteoporosis affects approximately 18.3% of older adults (1), and an estimated 54 million U.S. adults aged 50 years or older have osteoporosis or low bone mass (2, 3). Low bone mass thus defines a large and clinically consequential segment of the older population — and one in which a second, less conspicuous burden frequently coexists: depressive symptoms.

Depression and low bone mass are closely associated, with clinically important consequences for skeletal health. Meta-analyses have consistently linked depression to lower bone mineral density (BMD), with the largest deficits at the spine, hip, and femoral neck and stronger associations in women and in clinically diagnosed depression (4, 5). Prospectively, depression predicts accelerated bone loss and an approximately 24–26% higher risk of fracture across cohort studies (6, 7). Pooled individual-participant analyses have shown a graded association between depressive-symptom burden and incident cardiovascular disease (8). Depression has also been recognized as a risk factor for poor prognosis after acute coronary syndrome in an American Heart Association scientific statement (9). Lower BMD has also been associated with an increased risk of incident cardiovascular disease (10). These observations suggest that skeletal vulnerability, depressive symptoms, and cardiovascular risk may coexist in older adults. Yet depression in later life is routinely underdiagnosed and undertreated, partly because its somatic features overlap with those of coexisting medical illness and are too readily ascribed to aging itself (11, 12). Among older adults with low bone mass — who are already in regular clinical contact for skeletal or metabolic concerns — depressive symptoms may therefore go unrecognized precisely where their skeletal consequences matter most.

Depression is biologically heterogeneous, and a growing body of evidence identifies immunometabolic dysregulation — systemic low-grade inflammation together with disturbances of lipid and energy metabolism — as one biologically coherent dimension of the illness, present in an appreciable subset of patients and clustering with cardiometabolic comorbidity (13, 14). Contemporary cardiovascular research distinguishes residual inflammatory risk from residual cholesterol-related risk. Among statin-treated patients, high-sensitivity C-reactive protein (hs-CRP) was more strongly associated with subsequent cardiovascular events and mortality than low-density lipoprotein cholesterol (15). hs-CRP, a routinely measured marker of systemic inflammation, is repeatedly associated with depressive symptoms (16, 17), and the proinflammatory cytokines that accompany elevated CRP — tumor necrosis factor-α, interleukin-1, and interleukin-6 — drive osteoclast differentiation through the RANKL/osteoprotegerin axis and shift bone remodeling toward resorption (18, 19). Remnant cholesterol (RC), the cholesterol carried by triglyceride-rich lipoproteins (20), is likewise positively associated with depression in large population-based and prospective cohorts (21, 22) and inversely associated with spine BMD in U.S. adults (23). Crucially, these two axes are not independent: Mendelian randomization evidence indicates that elevated remnant cholesterol causally raises low-grade inflammation — a 1-mmol/L increment corresponding to approximately 28% higher CRP — whereas elevated low-density lipoprotein cholesterol does not (24), and their joint elevation confers greater risk than elevation of either marker alone (25).

The remnant cholesterol–inflammation index (RCII), defined as the product of calculated RC and cycle-specific CRP, was introduced in cardiovascular epidemiology to capture this shared metabolic–inflammatory burden in a single measure (26), and higher RCII has since been associated with stroke, diabetes, metabolic syndrome, cardiovascular events, and all-cause and cause-specific mortality across NHANES and Asian cohorts (27–30). A recent NHANES 2005–2010 analysis extended RCII to mental health, reporting a positive, approximately linear association between RCII and depression in the general U.S. adult population (31). Whether this association holds among older adults with low bone mass remains unknown. Two additional questions are whether calculated RC and cycle-specific CRP show distinguishable associations after mutual adjustment and whether the direction of the overall association is consistent in a separate clinical sample.

We examined the association between RCII and depressive-symptom screening positivity among adults aged ≥50 years with study-defined low bone mass using survey-weighted NHANES data. We characterized the dose–response shape and robustness of the association, evaluated the component-specific associations of calculated RC and cycle-specific CRP, tested heterogeneity by bone status, and assessed whether the direction of the primary association was consistent in a separate outcome-enriched clinical sample.

## 2 Methods

### 2.1 Study design and participants

This cross-sectional study used data from the 2005–2006, 2007–2008, and 2009–2010 cycles of the National Health and Nutrition Examination Survey (NHANES) and the 2017–March 2020 pre-pandemic release. NHANES uses a complex, multistage probability sampling design to represent the civilian, noninstitutionalized U.S. population. The source depression–symptom–bone phenotype sample included 7,569 participants with sufficient information for phenotype classification. We excluded 4,406 participants with neither study-defined low bone mass nor depressive-symptom screening positivity and 373 who screened positive without low bone mass, leaving 2,790 participants with study-defined low bone mass. We then excluded 614 participants who were younger than 50 years or had missing age and 142 with a missing or nonpositive pooled fasting-subsample weight. The remaining 2,034 participants had valid calculated remnant cholesterol (RC), cycle-specific C-reactive protein (CRP), and PHQ-9 data. After excluding 291 participants with incomplete Model 3 covariate data, the final NHANES analytic sample comprised 1,743 participants, including 139 with PHQ-9 scores of 10 or higher. A+ll participant counts are unweighted (Figure 1A). The study was reported in accordance with the STROBE statement.

**Figure 1:**
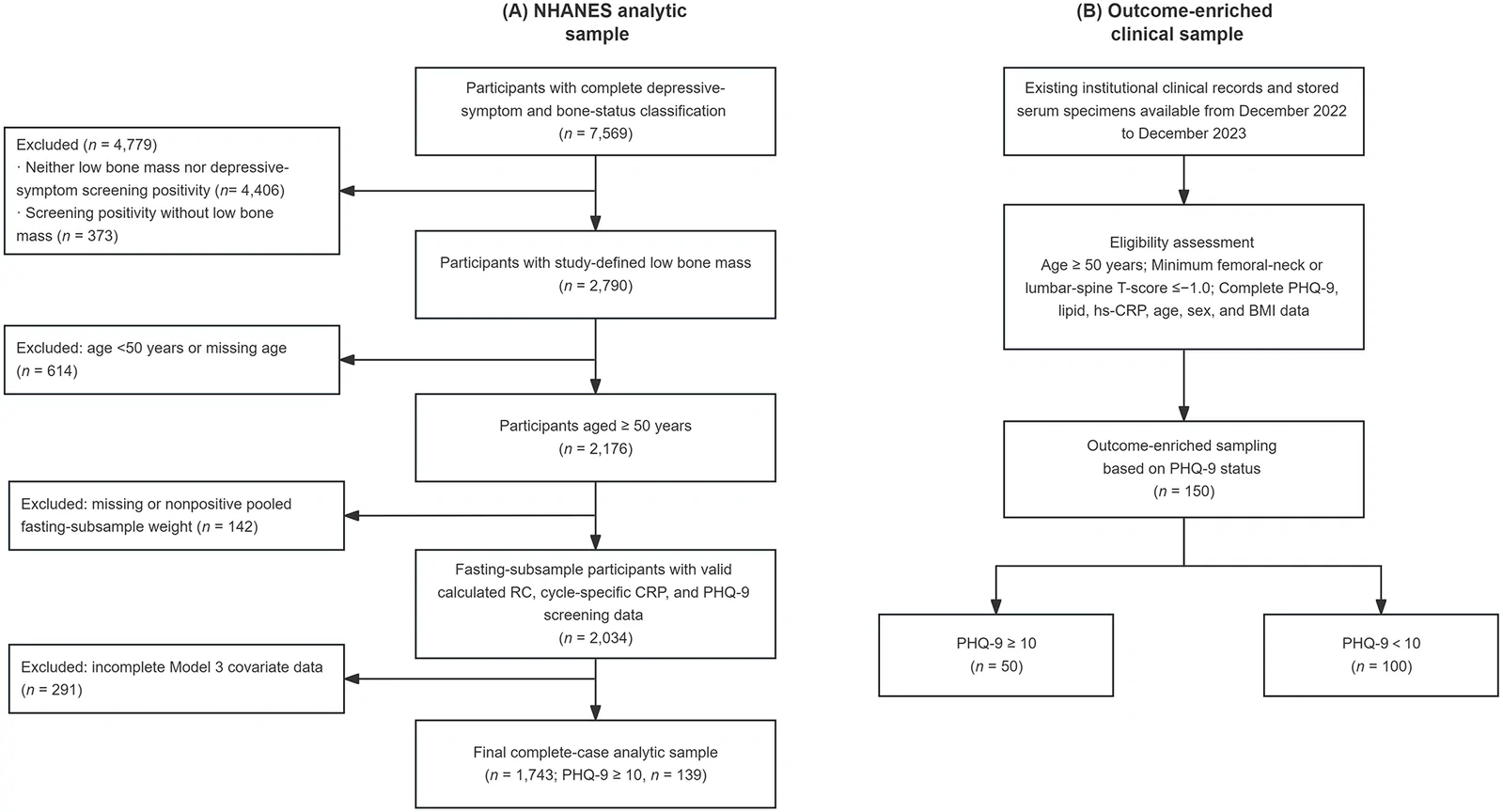
Participant selection for the NHANES analysis and outcome-enrichedclinical sample. **(A)** Selection of the primary NHANES sample. Of 7,569 participants with sufficient phenotype information, 1,743 adults aged ≥50 years with study-defined low bone mass, valid fasting-subsample data, and complete Model 3 covariates were included; 139 had depressive-symptom screening positivity. **(B)** Selection of the outcome-enriched clinical sample. Eligible patients were identified across existing institutional clinical data sources and stored serum specimens collected from December 2022 to December 2023 under uniform criteria. The analytical sample contained 50 participants with PHQ-9 ≥10 and 100 with PHQ-9 < 10; selection did not reference RCII, calculated RC, hs-CRP, or other lipid values. All counts are unweighted. BMI, body mass index; CRP, C-reactive protein; hs-CRP, high-sensitivity C-reactive protein; PHQ-9, Patient Health Questionnaire-9; RC, calculated remnant cholesterol.

The clinical component was a retrospective, single-center, outcome-enriched analysis of previously collected clinical information and stored serum specimens from Shenzhen Bao'an Chinese Medicine Hospital. The final analytical sample comprised 150 patients, including 100 with PHQ-9 scores < 10 and 50 with scores ≥10 (Figure 1B). Detailed source-cohort, timing, eligibility, measurement, and selection procedures are described in Section 2.7.

### 2.2 Assessment of low bone mass and depressive symptoms

Areal bone mineral density (BMD; g/cm^2^) was obtained from the NHANES DXA files. Femoral-neck BMD (DXXNKBMD) and total lumbar-spine L1–L4 BMD (DXXOSBMD) were used. Femoral-neck and lumbar-spine measurements were selected because these sites were available in both the NHANES and clinical samples. Total-hip BMD was not included; therefore, the resulting phenotype should be interpreted as a study-defined low-bone-mass classification rather than a comprehensive clinical DXA classification. Femoral-neck BMD was considered valid when the femur scan status was complete and valid (DXAFMRST = 1), the invalidity code was zero (DXXFMBCC = 0), and DXXNKBMD was positive and finite. Total lumbar-spine BMD was considered valid when the spine scan was complete with all vertebrae valid (DXASPNST = 1), the total-spine invalidity code was zero (DXXOSBCC = 0), and DXXOSBMD was positive and finite. When only one site was valid, the T-score from that site was used; participants with no valid T-score at either site were not classified.

Femoral-neck T-scores were calculated as (BMD−0.858)/0.120 using reference values from non-Hispanic White women aged 20–29 years in NHANES III (2). Total lumbar-spine T-scores were calculated as (BMD−1.047)/0.110 using the manufacturer reference for 30-year-old non-Hispanic White women (2). The same prespecified equations were applied to all eligible men and women in the NHANES analysis. The participant-level minimum was the lower available femoral-neck or total lumbar-spine T-score. Study-defined low bone mass was a minimum T-score ≤ -1.0 and therefore included both osteopenic and osteoporotic ranges; an osteoporosis-range minimum T-score was defined as ≤ -2.5. The clinical DXA system and its report-based interpretation rules are described in Section 2.7. Depressive symptoms were assessed with the nine-item Patient Health Questionnaire (PHQ-9; range, 0–27). The prespecified primary outcome was depressive-symptom screening positivity, defined as PHQ-9 ≥10. This threshold indicates clinically relevant symptom burden but does not establish a psychiatric diagnosis. The PHQ-9 total score was analyzed continuously as a secondary outcome.

### 2.3 Laboratory measurements and calculation of RCII

In NHANES 2005–2010, serum CRP (LBXCRP; mg/dL) was measured by latex-enhanced nephelometry. The lower reportable/detection limit was 0.02 mg/dL; values below the limit were released using the NHANES fill value (LLOD/√2). Values were converted to mg/L by multiplying by 10. In the 2017–March 2020 pre-pandemic release, hs-CRP (LBXHSCRP; mg/L) was measured by immunoturbidimetry; the lower limit of detection was 0.15 mg/L and below-limit results were released as LLOD/√2. Values above the assay range were handled through cycle-specific laboratory rerun or dilution procedures before public release. No additional cross-assay recalibration was applied. Instead, survey period was included in adjusted models and heterogeneity by survey period was examined. For fasting participants with triglycerides < 400 mg/dL, LDL-C was calculated using the Friedewald equation: LDL-C = total cholesterol–HDL-C–triglycerides/5. Calculated RC was defined as total cholesterol–HDL-C–LDL-C and was therefore algebraically equivalent to triglycerides/5 within this framework, apart from rounding. RCII was calculated as calculated RC (mg/dL) multiplied by the corresponding cycle-specific CRP measurement (mg/L).

Calculated RC, the cycle-specific CRP measurement, and RCII were natural-log transformed before analysis, and only positive, finite values were included. This transformation was prespecified because both components have positively skewed distributions, particularly CRP in the U.S. population (14, 32), and because the natural logarithm expresses the index as the sum of its components: ln(RCII) = ln(calculated RC) + ln(cycle-specific CRP). The transformation reduces the influence of extreme values and provides the scale used for the component-specific analyses described in Section 2.6. The use of calculated RC should not be interpreted as direct measurement of remnant lipoprotein particles.

### 2.4 Covariates

Covariates were selected *a priori* based on previous literature and their established associations with depressive symptoms, metabolic and inflammatory status, and skeletal health. Sociodemographic covariates included age, sex, race and ethnicity, educational attainment, marital status, family income-to-poverty ratio, and survey period. Lifestyle and clinical covariates included current smoking, a cycle-specific alcohol-use indicator, body mass index, diabetes, hypertension, and cardiovascular disease history. Age, family income-to-poverty ratio, and body mass index were modeled as continuous variables; the remaining covariates were treated as categorical variables. Current smoking was coded from SMQ020 and SMQ040 as current vs. noncurrent smoking. The alcohol indicator was coded from ALQ101 in 2005–2010 (≥12 drinks in any one year vs. no) and ALQ111 in 2017-March 2020 (ever vs. never having ≥1 drink), reflecting a change in the NHANES questionnaire across releases. In the locked Model 3 data, diabetes was based on self-reported physician diagnosis (DIQ010), and hypertension was based on self-reported physician diagnosis (BPQ020); HbA1c, antihypertensive medication use, and measured blood-pressure thresholds were not components of these final covariates. Cardiovascular disease was defined as a self-reported history of congestive heart failure, coronary heart disease, angina, heart attack, or stroke (MCQ160B-MCQ160F). Marital status was harmonized as married/cohabiting, never married, or widowed/divorced/separated, and education as below high school, high school/GED, or above high school. Refused, don't know, blank, and otherwise invalid responses were set to missing. The primary analysis used complete cases for the exposure, outcome, survey-design variables, and all Model 3 covariates, with no imputation. Survey period was categorized as 2005–2006, 2007–2008, 2009–2010, or 2017-March 2020. Detailed source-variable codes, category definitions, model reference levels, and missing-response rules are provided in Supplementary Table S7.

### 2.5 NHANES survey design and statistical analysis

All NHANES analyses incorporated masked variance strata, primary sampling units, and fasting-subsample weights. The 2-year fasting weights for each 2005–2010 cycle were multiplied by 2/9.2, and the 2017–March 2020 pre-pandemic fasting weight was multiplied by 3.2/9.2. Variances were estimated by Taylor series linearization. Analytic restrictions were implemented as survey subpopulations. The locked primary design had 72 design degrees of freedom. Complete-case analysis was used; missing data were not imputed.

Continuous characteristics were summarized using survey-weighted means and standard errors, except calculated RC and the cycle-specific CRP, which were summarized using weighted medians and interquartile ranges. Categorical characteristics were reported as unweighted counts and survey-weighted percentages. Mean-based comparisons used survey-weighted linear regression; calculated RC and the cycle-specific CRP measurement were tested on the natural-log scale; categorical variables were compared with Rao–Scott design-adjusted global tests.

Survey-weighted logistic regression was used to examine depressive-symptom screening positivity, with ln(RCII) modeled per 1 survey-weighted SD and in survey-weighted quartiles. Model 1 was unadjusted, Model 2 included sociodemographic factors and survey period, and Model 3 further included socioeconomic, lifestyle, anthropometric, and cardiometabolic covariates described above. Linear trend across quartiles was tested using the survey-weighted median ln(RCII) value of each quartile. Survey-weighted linear regression was used for continuous PHQ-9 scores. Confidence intervals and *P* values were based on model residual degrees of freedom.

Restricted cubic splines used knots at the weighted 10th, 50th, and 90th percentiles of ln(RCII) (1.766, 3.553, and 5.472), with the weighted median as the reference. Curves were displayed from the weighted 1st to 99th percentiles (0.358–6.838). The overall test jointly evaluated all spline basis terms, whereas the nonlinearity test evaluated only the nonlinear term while retaining the linear component.

Sensitivity analyses repeated Model 3 after restricting the cycle-specific CRP measurement to ≤ 10 mg/L and after excluding cardiovascular disease, diabetes, hypertension, or all three conditions. Effect modification by sex, age group, and BMI category was assessed using multiplicative interaction terms. For the three prespecified subgroup interaction tests for sex, age group, and BMI category, *P* values were additionally adjusted using the Benjamini–Hochberg false-discovery-rate procedure. Heterogeneity by survey period and bone status was evaluated using corresponding global interaction tests; bone-status analyses were conducted in the combined normal-bone and low-bone-mass sample, with stratum-specific estimates derived from the pooled interaction model. Statistical significance within individual subgroups was not used to infer effect modification.

### 2.6 Component-specific analysis

Component-specific analyses used the locked primary survey design and Model 3 covariates. Models included standardized ln(calculated RC), standardized ln(cycle-specific CRP), both components simultaneously, or standardized ln(RCII). A design-adjusted joint Wald test assessed whether both component coefficients were zero. Prespecified contrasts compared the two coefficients on the weighted-SD scale and tested the equal-coefficient constraint on the original logarithmic scale implied by ln(RCII) = ln(calculated RC) + ln(cycle-specific CRP). These tests assess statistical compatibility and do not establish equivalence, equal biological importance, synergy, attributable, or mediating effects, causal relationships, validation of equal weighting, or incremental value of RCII beyond CRP alone.

### 2.7 Retrospective outcome-enriched clinical sample

To assess directional consistency in a clinical setting, the outcome-enriched clinical sample was retrospectively assembled from existing institutional clinical records and stored serum specimens at Shenzhen Bao'an Chinese Medicine Hospital, Shenzhen, China. The source records and stored serum specimens used in the present analysis were collected from December 2022 to December 2023 and linked at the individual level before analysis. Uniform eligibility, timing, linkage, deduplication, and sampling procedures were applied across all sources.

Potentially eligible patients were identified across the available institutional sources using the same eligibility criteria. Patients were eligible if they: (1) were aged ≥50 years; (2) had low bone mass, defined as a minimum available femoral-neck or total lumbar-spine L1–L4 T-score ≤ -1.0 on DXA; (3) had completed the PHQ-9; (4) had fasting measurements of total cholesterol, HDL-C, and triglycerides; (5) had a stored serum specimen available for hs-CRP measurement; and (6) had complete data on age, sex, and BMI. Patients were excluded if the PHQ-9, DXA, lipid, and serum data could not be reliably linked to the same individual; if the interval between the required assessments exceeded 30 days; or if the serum volume or quality was insufficient for hs-CRP measurement. Duplicate encounters were removed before participant selection.

Following eligibility assessment and deduplication, the outcome-enriched clinical sample comprised all 50 eligible patients with PHQ-9 scores ≥10 and 100 eligible patients with PHQ-9 scores < 10. Participant selection was based on PHQ-9 screening status and fulfillment of the eligibility criteria, without reference to RCII, calculated RC, hs-CRP, lipid concentrations, or other covariates. This sampling strategy was used to assess the direction of the exposure–outcome association rather than to estimate the prevalence of screening positivity or absolute risk. No additional intervention or blood collection was performed specifically for the present retrospective analysis.

Clinical BMD was measured at the femoral neck and total lumbar spine (L1–L4) using a DPX Bravo dual-energy X-ray absorptiometry system (GE Medical Systems [China] Co., Ltd., China). Device-generated T-scores were based on the manufacturer-provided China reference database displayed in the DXA reports. Because all participants were aged ≥50 years, bone status in the present study was classified using T-scores. T-scores ≥-1.0, >-2.5 to < -1.0, and ≤ -2.5 were classified as normal bone mass, low bone mass, and osteoporosis-range bone mass, respectively. Clinical eligibility was determined using the lower available T-score from the femoral neck or total lumbar spine (L1–L4), consistent with the two-site minimum approach used in the NHANES analysis.

Fasting serum total cholesterol, HDL-C, and triglycerides were measured using a Chemray 2000 automated biochemical analyzer (Rayto Life and Analytical Sciences Co., Ltd., Shenzhen, China). LDL-C was calculated using the Friedewald equation because triglyceride concentrations were < 400 mg/dL in all included participants. Calculated remnant cholesterol (RC) was defined as total cholesterol minus HDL-C minus LDL-C. RCII was calculated as calculated RC (mg/dL) multiplied by serum hs-CRP (mg/L). RC, hs-CRP, and RCII were natural-log transformed where required, using the same definitions and units as in the NHANES analysis. Serum hs-CRP was measured using the High Sensitive Human C-Reactive Protein/hs-CRP ELISA Kit (catalog no. EK194-96; Multi Sciences [Lianke] Biotech Co., Ltd., Hangzhou, China). According to the manufacturer, the assay has a calibration range of 62.5–4,000 pg/mL, an analytical sensitivity of 0.44 pg/mL, and intra- and interassay coefficients of variation of 2.9%−5.5% and 3.0%−5.8%, respectively. Serum samples were initially diluted 2,000-fold and assayed in duplicate. Absorbance was measured at 450 nm. Samples exceeding the upper limit of the calibration range after the initial dilution were further diluted and re-assayed. Final hs-CRP concentrations were calculated using the total dilution factor, which was applied once, and converted to mg/L. The mean of duplicate measurements was used for subsequent analyses.

Clinical logistic regression models estimated the association between ln(RCII) and depressive-symptom screening positivity in crude and age-, sex-, and BMI-adjusted analyses. Continuous exposures were expressed per fixed NHANES survey-weighted SD, and sensitivity analysis was restricted to participants with hs-CRP ≤ 10 mg/L. Given the outcome-enriched sampling design, the clinical analysis was interpreted as supportive directional corroboration. Estimates from the NHANES and clinical samples were presented side by side using the same fixed exposure scale but were not statistically pooled.

## 3 Results

### 3.1 Participant selection and baseline characteristics

The source phenotype sample included 7,569 participants. Sequential restriction to study-defined low bone mass, age 50 years or older, a positive fasting-subsample weight, valid calculated RC, cycle-specific CRP, and PHQ-9 data, and complete Model 3 covariates yielded 1,743 NHANES participants; 139 had depressive-symptom screening positivity (Figure 1A). The unweighted ln(RCII) quartile counts were 373, 416, 470, and 484. Across increasing quartiles, family income-to-poverty ratio declined and body mass index increased, while calculated RC and cycle-specific CRP measurement showed the expected exposure gradients (Table 1). Characteristics of the primary NHANES sample according to depressive-symptom screening status are presented in Supplementary Table S1.

**Table 1:** Survey-weighted characteristics of adults aged ≥50 years with study-defined low bone mass according to weighted quartiles of ln(RCII).

Characteristic	Overall	Q1	Q2	Q3	Q4	*P* value
No. of participants, unweighted	1743	373	416	470	484	—
Age, years	64.69 (0.33)	62.48 (0.70)	65.25 (0.63)	65.81 (0.61)	65.24 (0.58)	0.004
Family income-to-poverty ratio	3.20 (0.06)	3.52 (0.08)	3.40 (0.11)	2.97 (0.08)	2.88 (0.12)	< 0.001
Body mass index, kg/m^2^	27.09 (0.16)	24.23 (0.27)	26.84 (0.32)	27.77 (0.39)	29.52 (0.35)	< 0.001
Minimum T-score	−1.87 (0.02)	−1.91 (0.04)	−1.92 (0.05)	−1.86 (0.03)	−1.77 (0.04)	0.039
Calculated remnant cholesterol, mg/dL	20.00 [15.00, 29.00]	14.00 [10.00, 19.00]	20.00 [15.00, 27.00]	23.00 [17.00, 31.00]	28.00 [21.00, 40.00]	< 0.001
Cycle-specific CRP, mg/L	1.60 [0.70, 3.63]	0.45 [0.30, 0.66]	1.06 [0.80, 1.50]	2.40 [1.80, 3.30]	6.62 [4.30, 11.59]	< 0.001
ln(RCII)	3.56 (0.05)	1.77 (0.06)	3.09 (0.02)	4.01 (0.01)	5.36 (0.04)	< 0.001
Depressive-symptom screening positivity	139 (6.0%)	20 (3.5%)	24 (3.5%)	37 (7.2%)	58 (9.9%)	< 0.001
Female sex	1116 (69.6%)	228 (71.7%)	263 (67.2%)	303 (67.7%)	322 (71.8%)	0.561
Race/ethnicity						0.119
Mexican American	246 (4.4%)	39 (2.9%)	53 (4.1%)	70 (5.0%)	84 (5.6%)	
Other Hispanic	164 (4.0%)	29 (2.8%)	34 (3.8%)	51 (5.0%)	50 (4.3%)	
Non-Hispanic White	996 (80.3%)	208 (81.3%)	247 (81.0%)	263 (77.8%)	278 (81.1%)	
Non-Hispanic Black	199 (5.1%)	46 (4.5%)	46 (5.1%)	58 (5.7%)	49 (5.2%)	
Other race/multiracial	138 (6.2%)	51 (8.6%)	36 (6.0%)	28 (6.5%)	23 (3.8%)	
Educational attainment						< 0.001
Above high school	817 (56.6%)	216 (66.2%)	214 (60.5%)	193 (49.4%)	194 (50.5%)	
High school or GED	446 (27.7%)	86 (25.2%)	102 (25.3%)	134 (32.9%)	124 (27.5%)	
Below high school	480 (15.6%)	71 (8.7%)	100 (14.2%)	143 (17.7%)	166 (22.0%)	
Marital status						0.901
Married or cohabiting	1014 (62.6%)	235 (65.8%)	251 (62.5%)	266 (61.8%)	262 (60.4%)	
Never married	84 (3.8%)	23 (4.0%)	24 (4.0%)	15 (3.6%)	22 (3.6%)	
Widowed/divorced/separated	645 (33.6%)	115 (30.3%)	141 (33.6%)	189 (34.7%)	200 (36.1%)	
Current smoking	253 (14.4%)	35 (10.7%)	40 (10.3%)	77 (17.3%)	101 (19.2%)	0.009
Alcohol use	1256 (77.3%)	293 (81.8%)	292 (76.3%)	325 (73.6%)	346 (77.6%)	0.174
Diabetes	279 (11.1%)	48 (7.1%)	65 (9.3%)	78 (12.2%)	88 (15.7%)	< 0.001
Hypertension	874 (45.0%)	152 (36.5%)	208 (45.7%)	246 (49.1%)	268 (48.8%)	0.014
Cardiovascular disease	335 (16.4%)	44 (11.4%)	80 (15.7%)	96 (17.1%)	115 (21.4%)	0.051

Continuous variables are presented as survey-weighted mean (standard error), except calculated remnant cholesterol and the cycle-specific CRP, which are presented as survey-weighted median [25th, 75th percentiles]. Categorical variables are presented as unweighted number (survey-weighted percentage). The ln(RCII) quartile groups were defined using survey-weighted cut points of 2.630, 3.553, and 4.482. *P* values are two-sided and unadjusted. Mean-based continuous variables were compared using survey-weighted linear regression; calculated remnant cholesterol and the cycle-specific CRP measurement were natural-log transformed for hypothesis testing. Categorical variables were evaluated using Rao-Scott design-adjusted global tests. All estimates incorporated pooled fasting-subsample weights, masked variance strata, and primary sampling units. CRP, C-reactive protein; PHQ-9, Patient Health Questionnaire-9; RCII, remnant cholesterol-inflammation index; SE, standard error.

All 150 participants in the final outcome-enriched clinical sample met the age and low-bone-mass criteria and had complete exposure, outcome, and adjustment-variable data; therefore, no clinical participant was excluded after selection into the analytical dataset. The sample included 100 participants with PHQ-9 scores < 10 and 50 with scores ≥10 (Figure 1B). These proportions reflect the prespecified outcome-enriched sampling scheme and must not be interpreted as the prevalence of depressive-symptom screening positivity in a clinical low-bone-mass population.

### 3.2 Association of ln(RCII) with depressive-symptom screening positivity

Higher ln(RCII) was associated with greater odds of depressive-symptom screening positivity across all three models. In the fully adjusted model, each 1 survey-weighted SD increment in ln(RCII) was associated with 52% higher odds of screening positivity (OR, 1.52; 95% CI, 1.15–2.00; *P* = 0.004). Compared with quartile 1, the fully adjusted ORs were 1.24 (95% CI, 0.56–2.74; *P* = 0.586) for quartile 2, 2.13 (95% CI, 0.89–5.10; *P* = 0.089) for quartile 3, and 3.00 (95% CI, 1.40–6.43; *P* = 0.005) for quartile 4. The trend across survey-weighted quartiles was statistically significant (*P* for trend = 0.002; Table 2).

**Table 2:** Survey-weighted associations between ln(RCII) and depressive-symptom screening positivity.

ln(RCII)	Model 1		Model 2		Model 3	
	OR (95% CI)	*P* value	OR (95% CI)	*P* value	OR (95% CI)	*P* value
Continuous, per weighted SD	1.62 (1.27–2.07)	< 0.001	1.58 (1.21–2.07)	0.001	1.52 (1.15–2.00)	0.004
Quartile 1	Reference	—	Reference	—	Reference	—
Quartile 2 vs 1	1.00 (0.47–2.12)	0.994	1.02 (0.47–2.23)	0.950	1.24 (0.56–2.74)	0.586
Quartile 3 vs 1	2.14 (1.01–4.52)	0.048	2.09 (0.93–4.67)	0.073	2.13 (0.89–5.10)	0.089
Quartile 4 vs 1	3.04 (1.65–5.63)	< 0.001	2.98 (1.56–5.69)	0.001	3.00 (1.40–6.43)	0.005
*P* for trend	1.46 (1.22–1.76)	< 0.001	1.45 (1.20–1.75)	< 0.001	1.42 (1.14–1.76)	0.002

Odds ratios and 95% confidence intervals were estimated using survey-weighted logistic regression. Model 1 was unadjusted. Model 2 adjusted for age, sex, race/ethnicity, educational attainment, marital status, and survey period. Model 3 additionally adjusted for family income-to-poverty ratio, current smoking, alcohol use, body mass index, diabetes, hypertension, and cardiovascular disease history. *P* for trend was calculated by assigning participants the survey-weighted median ln(RCII) value of the corresponding quartile and modeling this value continuously. The weighted SD of ln(RCII) was 1.404; weighted quartile cut points were 2.630, 3.553, and 4.482. CI, confidence interval; OR, odds ratio; PHQ-9, Patient Health Questionnaire-9; RCII, remnant cholesterol–inflammation index; SD, standard deviation.

In the secondary analysis of symptom severity, each 1 survey-weighted SD increment in ln(RCII) was associated with a 0.38-point higher PHQ-9 total score after full adjustment (β, 0.38; 95% CI, 0.13–0.63; *P* = 0.004). Compared with quartile 1, quartile 4 was associated with a 0.80-point higher PHQ-9 score (β, 0.80; 95% CI, 0.17–1.44; *P* = 0.015), with a significant trend across quartiles (*P* for trend = 0.008; Supplementary Table S2).

### 3.3 Dose–response association

In the fully adjusted restricted cubic spline model, ln(RCII) was associated with depressive-symptom screening positivity (*P* for overall = 0.012), with no strong evidence of nonlinearity (*P* for nonlinearity = 0.874; Figure 2). The estimated OR increased above the reference median as ln(RCII) increased. Confidence intervals widened at the lower and upper ends of the exposure distribution, indicating reduced precision at the extremes. The spline analysis did not provide strong evidence of departure from linearity on the log-odds scale and did not identify a distinct clinical threshold. Results from Models 1 and 2 were consistent with the fully adjusted analysis (Supplementary Figure S2).

**Figure 2:**
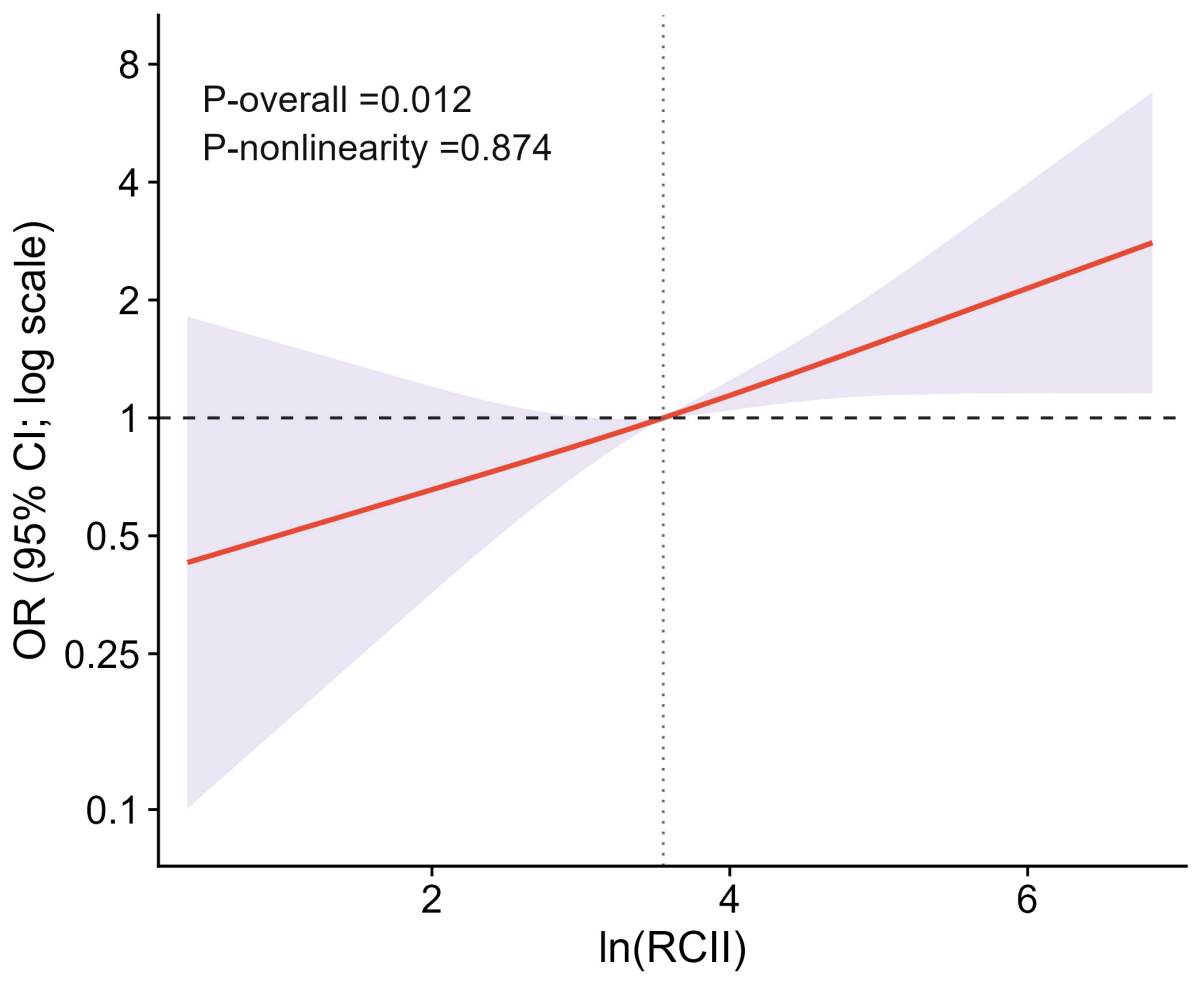
Fully adjusted association between ln(RCII) and depressive-symptom screening positivity. Survey-weighted restricted cubic spline analysis was conducted among 1,743 adults aged ≥50 years with study-defined low bone mass. The solid line represents adjusted odds ratios (ORs), and the shaded area represents 95% confidence intervals (CIs). Knots were placed at the survey-weighted 10th, 50th, and 90th percentiles of ln(RCII) (1.766, 3.553, and 5.472, respectively), with the weighted median (3.553) as the reference. The vertical dotted line indicates the reference median, and the horizontal dashed line indicates an OR of 1. The curve is displayed from the survey-weighted 1st to 99th percentiles, and the y-axis is on a logarithmic scale. The test for nonlinearity did not provide strong evidence of departure from linearity (*P* = 0.874). The model was adjusted for age, sex, race and ethnicity, educational attainment, marital status, survey period, family income-to-poverty ratio, current smoking, alcohol use, body mass index, diabetes, hypertension, and cardiovascular disease history. CI, confidence interval; OR, odds ratio; RCII, remnant cholesterol–inflammation index.

### 3.4 Component-specific and composite-index analyses

When examined separately in fully adjusted models, standardized ln(calculated RC) was not significantly associated with depressive-symptom screening positivity (OR, 1.18; 95% CI, 0.92–1.51; *P* = 0.178), whereas standardized ln(cycle-specific CRP) was associated with higher odds of screening positivity (OR, 1.45; 95% CI, 1.13–1.85; *P* = 0.004). In the mutually adjusted model, the OR was 1.14 for standardized ln(calculated RC; 95% CI, 0.89–1.47; *P* = 0.284) and 1.44 for standardized ln(cycle-specific CRP; 95% CI, 1.12–1.84; *P* = 0.005). The corresponding OR for standardized ln(RCII) was 1.52 (95% CI, 1.15–2.00; *P* = 0.004; Table 3).

Table 3Component-specific and composite-index associations with depressive-symptom screening positivity.

**Table:** 

Panel A. Component-specific and composite-index models			
Model	Exposure	Adjusted OR (95% CI)	*P* value
Model A: calculated RC alone	ln(calculated RC), per 1 survey-weighted SD	1.18 (0.92–1.51)	0.178
Model B: cycle-specific CRP alone	ln(cycle-specific CRP), per 1 survey-weighted SD	1.45 (1.13–1.85)	0.004
Model C: calculated RC + cycle-specific CRP	ln(calculated RC), mutually adjusted, per 1 survey-weighted SD	1.14 (0.89–1.47)	0.284
	ln(cycle-specific CRP), mutually adjusted, per 1 survey-weighted SD	1.44 (1.12–1.84)	0.005
Model D: RCII	ln(RCII), per 1 survey-weighted SD	1.52 (1.15–2.00)	0.004

**Table:** 

Panel B. Joint and contrast tests from the two-component model			
Test	Estimate (95% CI)	Test statistic	*P* value
Joint association of standardized ln(calculated RC) and ln(cycle-specific CRP)	—	*F* (2, 50) = 4.60	0.015
Standardized coefficient difference: β[z{ln(calculated RC)}] – β[z{ln(cycle-specific CRP)}]	−0.228 (−0.563 to 0.108)	*t* (50) = −1.36	0.179
Original-log-scale coefficient difference: β[ln(calculated RC)] – β[ln(cycle-specific CRP)]	−0.036 (−0.553 to 0.481)	*t* (50) = −0.14	0.890

All models were adjusted for Model 3 covariates. Standardized exposures were expressed per 1 survey-weighted SD. Model C included standardized ln(calculated RC) and ln(cycle-specific CRP) simultaneously. The joint Wald test evaluated whether both component coefficients were zero. The standardized coefficient contrast compared the two associations after scaling each component to 1 survey-weighted SD. The original-log-scale contrast tested the equal-coefficient constraint implied by ln(RCII) = ln(calculated RC) + ln(cycle-specific CRP).

The two component coefficients were jointly associated with screening positivity [*F*_(2, 50)_ = 4.60; *P* = 0.015]. However, the standardized coefficient difference between ln(calculated RC) and ln(cycle-specific CRP) was not statistically significant (difference, −0.228; 95% CI, −0.563 to 0.108; *P* = 0.179). The original-log-scale coefficient difference was also nonsignificant (difference, −0.036; 95% CI, −0.553 to 0.481; *P* = 0.890). These findings do not establish equivalence or equal biological importance, do not validate equal weighting in RCII, and do not demonstrate incremental value of RCII beyond CRP alone.

### 3.5 Supportive clinical corroboration

All 150 participants in the final outcome-enriched clinical sample had complete exposure, outcome, and covariate data. The sample comprised all 50 eligible patients with PHQ-9 scores ≥10 and 100 eligible patients with PHQ-9 scores < 10, as described in Section 2.7 (Figure 1B). Characteristics of the 150 participants included in the outcome-enriched clinical sample are summarized in Table 4. Of these, 100 had PHQ-9 scores < 10 and 50 had PHQ-9 scores ≥10. Participants with depressive-symptom screening positivity had higher hs-CRP, ln(RCII), and LDL-C values and lower HDL-C concentrations than those without screening positivity. Age, sex, body mass index, minimum T-score, and RC did not differ significantly between groups. Because sampling was outcome-enriched according to PHQ-9 screening status, these comparisons were descriptive, and the observed group proportions should not be interpreted as population prevalence. In the clinical sample, higher ln(RCII) was associated with greater odds of depressive-symptom screening positivity. Each 1 fixed NHANES survey-weighted SD increment in ln(RCII) was associated with an OR of 3.14 (95% CI, 1.86–5.28; *P* < 0.001) in the crude model. After adjustment for age, sex, and BMI, the association remained essentially unchanged (OR, 3.29; 95% CI, 1.92–5.64; *P* < 0.001). Restriction to participants with hs-CRP concentrations ≤ 10 mg/L yielded a similar estimate (*N* = 141; 44 screening-positive participants; OR, 3.29; 95% CI, 1.83–5.94; *P* < 0.001; Table 5; Figure 3B).

**Table 4:** Characteristics of the supportive clinical corroboration sample by depressive-symptom screening status.

Characteristic	Overall (*N* = 150)	PHQ-9 < 10 (*n* = 100)	PHQ-9 ≥10 (*n* = 50)	*P* value
Age, years	62.99 (7.13)	63.23 (6.65)	62.50 (8.06)	0.582
Female sex	101 (67.3)	67 (67.0)	34 (68.0)	1.000
Body mass index, kg/m^2^	25.37 (2.85)	25.22 (2.71)	25.67 (3.11)	0.393
Minimum T-score	−2.30 (0.66)	−2.25 (0.71)	−2.40 (0.56)	0.150
Osteoporosis-range minimum T-score	49 (32.7)	29 (29.0)	20 (40.0)	0.199
PHQ-9 total score	4.00 [3.00, 12.00]	4.00 [2.00, 4.00]	13.00 [12.00, 15.75]	—
Total cholesterol, mg/dL	199.97 [181.14, 221.17]	200.74 [180.05, 221.77]	198.80 [188.38, 215.93]	0.976
HDL-C, mg/dL	53.11 [42.29, 64.72]	57.99 [48.97, 67.14]	39.55 [32.30, 52.50]	< 0.001
LDL-C, mg/dL	122.69 [104.93, 150.72]	120.25 [97.48, 146.72]	133.87 [114.11, 154.88]	0.034
Calculated remnant cholesterol, mg/dL	20.65 [16.54, 26.74]	19.99 [16.23, 26.33]	22.00 [17.99, 27.76]	0.134
hs-CRP, mg/L	2.29 [1.17, 5.04]	1.65 [0.78, 4.15]	3.92 [2.17, 6.52]	< 0.001
ln(RCII)	3.84 (1.20)	3.53 (1.19)	4.48 (0.93)	< 0.001

All 150 included patients met the prespecified age and low-bone-mass eligibility criteria. Values are unweighted mean (SD), median [IQR], or *n* (%), as appropriate. *P* values were obtained using Welch t tests, Mann–Whitney U tests, or Fisher exact tests. Because recruitment was fixed by screening status, the group proportions do not estimate population prevalence. No between-group *P* value was reported for the PHQ-9 total score because the screening groups were defined using the PHQ-9 threshold. CRP, C-reactive protein; hs-CRP, high-sensitivity C-reactive protein; PHQ-9, Patient Health Questionnaire-9; RC, calculated remnant cholesterol; RCII, remnant cholesterol–inflammation index.

**Table 5:** Supportive directional corroboration of the association between ln(RCII) and depressive-symptom screening positivity.

Analysis	*N*	Events	Adjustment	OR per fixed NHANES SD (95% CI)	*P* value
Outcome-enriched clinical sample, crude	150	50	Unadjusted	3.14 (1.86–5.28)	< 0.001
Outcome-enriched clinical sample, age/sex/BMI adjusted	150	50	Age, sex, and BMI	3.29 (1.92–5.64)	< 0.001
hs-CRP ≤ 10 mg/L, age/sex/BMI adjusted	141	44	Age, sex, and BMI	3.29 (1.83–5.94)	< 0.001

The prespecified primary clinical analysis was the age-, sex-, and BMI-adjusted model among all 150 participants (50 screening-positive participants). ln(RCII) was expressed per fixed NHANES survey-weighted SD (1.403742). Because the clinical sample was outcome-enriched according to PHQ-9 screening status, the observed screening-positive proportion does not estimate population prevalence. The sample was used to examine directional consistency of the exposure–outcome odds-ratio association rather than to estimate population absolute risk. Sample-specific estimates were not directly compared or statistically pooled because of differences in sampling mechanism and covariate adjustment.

**Figure 3:**
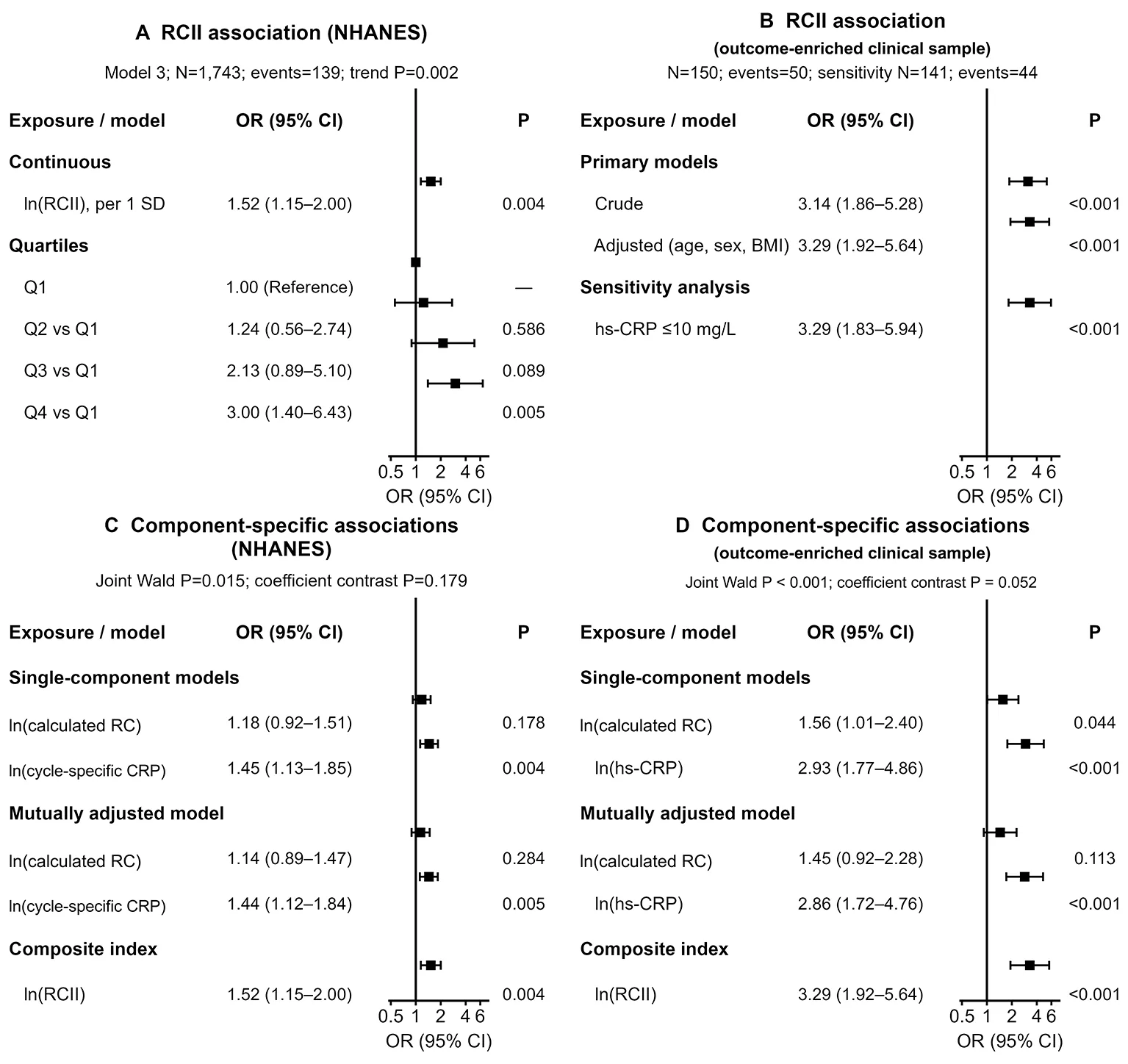
Cross-sample directional corroboration and component-specific associations of RCII with depressive-symptom screening positivity. **(A)** Survey-weighted associations of ln-transformed remnant cholesterol–inflammation index [ln(RCII)] with depressive-symptom screening positivity in the NHANES primary sample, adjusted for Model 3 covariates and including the continuous and quartile-based analyses. **(B)** Corresponding crude, age-, sex-, and BMI-adjusted, and hs-CRP ≤ 10 mg/L sensitivity estimates in the outcome-enriched clinical sample, adjusted for age, sex, and BMI. **(C)** Component-specific associations of ln(calculated RC), ln(cycle-specific CRP), and ln(RCII) in NHANES, adjusted for Model 3 covariates. **(D)** Corresponding component-specific associations of ln(calculated RC), ln(hs-CRP), and ln(RCII) in the outcome-enriched clinical sample, adjusted for age, sex, and BMI. Continuous exposures were expressed per the corresponding fixed NHANES survey-weighted SD. The vertical reference line indicates an OR of 1. Joint Wald tests evaluated whether both component coefficients were zero, and coefficient contrasts compared their standardized associations of ln(calculated RC) and the corresponding inflammatory component within each sample. Sample-specific estimates were displayed side by side and were not statistically pooled because of differences in sampling mechanism and covariate adjustment. BMI, body mass index; CI, confidence interval; CRP, C-reactive protein; hs-CRP, high-sensitivity C-reactive protein; NHANES, National Health and Nutrition Examination Survey; OR, odds ratio; RC, calculated remnant cholesterol; RCII, remnant cholesterol–inflammation index; SD, standard deviation.

When expressed on the fixed NHANES SD scale, the clinical estimate was directionally consistent with the association observed in NHANES (Figures 3A, B). Because the samples differed in sampling mechanism, source population, and covariate adjustment, sample-specific estimates were displayed side by side and were not statistically pooled; the larger clinical OR should not be interpreted as evidence of a stronger underlying effect.

Component-specific analyses showed a directionally similar statistical pattern across samples (Figures 3C, D). In the clinical sample, ln(hs-CRP) was associated with screening positivity when modeled separately (OR per fixed NHANES SD, 2.93; 95% CI, 1.77–4.86; *P* < 0.001) and remained associated after mutual adjustment for ln(calculated RC; OR, 2.86; 95% CI, 1.72–4.76; *P* < 0.001). In contrast, the association for ln(calculated RC) was attenuated after mutual adjustment for ln(hs-CRP) (OR, 1.45; 95% CI, 0.92–2.28; *P* = 0.113). The two component coefficients were jointly associated with screening positivity (Wald χ^2^(2) = 18.94; *P* < 0.001), whereas the standardized coefficient contrast between ln(calculated RC) and ln(hs-CRP) was not statistically significant (β difference, −0.682; 95% CI, −1.370 to 0.006; *P* = 0.052; Supplementary Table S3). These results provide directional corroboration only and do not establish component equivalence, unequal biological importance, or incremental clinical value beyond hs-CRP.

### 3.6 Sensitivity and subgroup analyses

The association between ln(RCII) and depressive-symptom screening positivity remained positive across all sensitivity analyses. The adjusted OR per 1 fixed NHANES survey-weighted SD increment in ln(RCII) was 1.56 (95% CI, 1.07–2.28) after restricting the sample to participants with cycle-specific CRP concentrations ≤ 10 mg/L, 1.63 (95% CI, 1.19–2.22) after excluding participants with cardiovascular disease, 1.49 (95% CI, 1.10–2.02) after excluding participants with diabetes, and 1.56 (95% CI, 1.12–2.16) after excluding participants with hypertension. After excluding participants with any of cardiovascular disease, diabetes, or hypertension, the corresponding OR was 1.41 (95% CI, 1.03–1.91). Quartile-based estimates were also directionally consistent across the restricted samples (Supplementary Table S4).

There was no evidence of effect modification by sex (*P* for interaction = 0.298), age group (*P* for interaction = 0.440), or BMI category (*P* for interaction = 0.372). The three interaction tests remained nonsignificant after Benjamini–Hochberg false-discovery-rate adjustment (all FDR-adjusted *P* = 0.440; Supplementary Figure S1).

### 3.7 Survey-period and bone-status heterogeneity

There was no evidence that the association between ln(RCII) and depressive-symptom screening positivity differed across NHANES survey periods (design-adjusted *F*(3,48) = 0.52; *P* for interaction = 0.674). Period-specific adjusted ORs derived from the pooled interaction model ranged from 1.27 to 1.81 per fixed NHANES SD increment in ln(RCII) (Supplementary Table S5).

In the combined normal-bone and low-bone-mass sample, the adjusted OR per fixed SD increment in ln(RCII) was 1.33 (95% CI, 1.03–1.73) among participants with normal bone status and 1.37 (95% CI, 1.04–1.79) among those with low bone mass. The ratio of ORs was 1.02 (95% CI, 0.73–1.45; *P* for interaction = 0.888). No interaction by bone status was detected when PHQ-9 total score was analyzed as a continuous outcome (P for interaction = 0.692; Supplementary Table S6).

## 4 Discussion

In this survey-weighted analysis of 1,743 adults aged 50 years or older with study-defined low bone mass, higher ln(RCII) was associated with greater odds of depressive-symptom screening positivity and higher PHQ-9 scores. Each survey-weighted standard-deviation increment in ln(RCII) corresponded to 52% higher odds of a positive screen, and participants in the highest quartile had approximately threefold higher odds than those in the lowest, with a significant trend across quartiles and no strong evidence of nonlinearity. In mutually adjusted component models, cycle-specific CRP retained an association with screening positivity whereas calculated RC did not, although formal coefficient contrasts did not establish a statistically stronger association for CRP. The association was directionally consistent in sensitivity analyses that restricted CRP to ≤ 10 mg/L or excluded participants with cardiovascular disease, diabetes, or hypertension. The direction of association was also consistent in the outcome-enriched clinical sample, although the sample-specific estimates should not be interpreted as comparable population effects. Formal interaction tests provided no strong evidence of heterogeneity by sex, age, body mass index, survey period, or bone status.

The clinical analysis was designed as supportive directional corroboration, not as a population-representative replication or a test of transportability. The same direction of association was observed in a distinct low-bone-mass sample, but differences in sampling, covariate adjustment, and laboratory procedures limit direct comparison of the two estimates. This finding is compatible with previous population studies linking remnant cholesterol, CRP, or RCII with depressive symptoms (21, 22, 31, 33); the present study extends this evidence to adults with study-defined low bone mass, a group in which depression and lower bone mineral density are known to co-occur (4, 5).

Potential biological mechanisms are presented here as hypotheses rather than as explanations established by the present data. Inflammatory signaling has been proposed to influence both serotonergic metabolism and bone remodeling through cytokine, kynurenine, and RANKL/OPG pathways (18, 19, 34, 35), and lipid dysregulation has been linked experimentally to serotonergic signaling, blood–brain-barrier function, neuroinflammation, and altered osteoblast and osteoclast activity (36–39). These pathways provide biological plausibility for an association between a metabolic–inflammatory composite and depressive-symptom burden in adults with low bone mass, but none of them was measured in this cross-sectional analysis.

Calculated RC and CRP may be correlated markers of related metabolic–inflammatory processes: genetic evidence has linked elevated remnant cholesterol to low-grade inflammation, and their dual elevation has been associated with adverse cardiovascular outcomes (24, 25), but the present calculated RC was algebraically equivalent to triglycerides/5 and did not directly quantify remnant lipoprotein particles. In this dataset, RCII is therefore effectively a triglyceride–CRP product scaled by a constant. The study did not measure cytokines, vascular disease, remnant lipoprotein particles, or bone-turnover markers, and it did not test RC-by-CRP interaction or mediation. Accordingly, RCII should be interpreted as a calculated composite correlate rather than as evidence of a self-reinforcing biological loop or a specific mechanistic pathway.

This interpretation is consistent with the component analysis. The CRP component retained an association after mutual adjustment, whereas calculated RC did not, but direct statistical comparison did not confirm a difference between their associations (40); the nonsignificant contrast most likely reflects limited precision and should not be read as proof of equivalence. Likewise, the equal-coefficient constraint implied by ln(RCII) = ln(calculated RC) + ln(cycle-specific CRP) was not rejected, but this does not validate equal biological or clinical weighting. Shared variance between calculated RC and CRP may reduce precision in mutually adjusted models, and the nonsignificant calculated-RC association should not be interpreted as evidence that this component has no association with depressive symptoms.

The strengths of this study follow directly from its principal findings. First, the central association was estimated within a rigorous survey-analytic framework (NHANES sampling weights, complex-survey variance estimation, a fixed exposure scale, and quartile thresholds held constant across analyses), supporting population-relevant estimation of the observed association. Second, evaluating both binary screening positivity and continuous PHQ-9 severity showed that the association was not dependent on a single cut point. Third, resolving RCII into its lipid and inflammatory constituents through formal component-coefficient contrasts moved the analysis beyond a black-box composite while preserving the distinction between a significant component association and a nonsignificant coefficient contrast. Finally, a directionally concordant analysis in a separate clinical sample provides supportive corroboration, subject to the provenance and sampling limitations described below.

Several limitations temper these findings. The cross-sectional NHANES design precludes inference about temporality or causality and does not exclude reverse pathways. Residual confounding may remain because medication use, diet, physical activity, acute illness, chronic pain, and psychosocial factors were not comprehensively characterized. PHQ-9 screening positivity indicates clinically relevant symptom burden, and although a threshold of 10 or above shows acceptable accuracy against diagnostic interviews (41), a positive screen is not a diagnosis of major depressive disorder. Because LDL-C was estimated using the Friedewald equation (42), calculated RC was algebraically equivalent to triglycerides divided by five when lipid concentrations were expressed in mg/dL; RCII therefore effectively represented a scaled triglyceride–CRP product rather than a direct measure of remnant lipoprotein cholesterol. CRP measurements differed across NHANES cycles and between NHANES and the clinical sample, which may have introduced assay-related heterogeneity. The study did not formally test RC-by-CRP interaction or mediation, and it did not evaluate predictive performance. Estimates were less precise at the exposure extremes, and the number of screening-positive events limited power for interaction and nonlinearity testing.

Finally, the clinical sample was derived from a single-center, outcome-enriched design and was adjusted for a limited set of covariates. Differences between the NHANES and clinical samples in source population, participant selection, assessment timing, DXA reference standards, and laboratory procedures limit direct cross-sample comparison. Accordingly, the larger odds ratio observed in the clinical sample should not be interpreted as evidence of a stronger underlying association, and the observed outcome proportion should not be interpreted as a population prevalence estimate.

From a public-health perspective, these findings identify a metabolic–inflammatory correlate of depressive-symptom burden among adults aged ≥50 years with study-defined low bone mass. Because calculated RC can be derived from a standard lipid panel, RCII can be readily calculated when comparable CRP measurements are available. The present evidence, however, does not justify routine RCII-based depressive-symptom screening, a diagnostic threshold, or treatment decisions. Prospective studies using natural outcome sampling, repeated exposure measurement, directly characterized remnant lipoproteins, and clinically confirmed depressive disorders are required before clinical application can be considered.

## 5 Conclusion

Among adults aged ≥50 years with study-defined low bone mass, higher RCII was associated with greater depressive-symptom burden. The null bone-status interaction and similar estimates in participants with normal and low bone mass do not indicate that this association is unique to or stronger in the low-bone-mass subgroup. CRP retained an association after mutual adjustment, whereas calculated RC did not; however, the direct component contrast was imprecise and nonsignificant, and incremental value beyond CRP alone was not established. The outcome-enriched clinical sample provided supportive directional corroboration. These findings do not establish causality or clinical predictive utility and require confirmation in prospective multicenter studies.

## Data Availability

The NHANES datasets analyzed in this study are publicly available in the CDC repository at https://www.cdc.gov/nchs/nhanes/index.html. De-identified data from the supportive clinical sample are available from the corresponding author upon reasonable request and after institutional review and approval.
