# Supplementary material for: Remnant cholesterol–inflammation index and depressive symptoms in middle-aged and older adults with study-defined low bone mass: component-specific analysis and supportive clinical corroboration
Source: Front Public Health. 2026 Oct 9;14:1976857. doi: 10.3389/fpubh.2026.1976857 (PMC13698836; doi:10.3389/fpubh.2026.1976857)
Supplement: Supplementary file 1 [file Table_1.docx]

**Supplementary Methods**

**S1. NHANES survey weighting and CRP measurements**

For NHANES 2005–2010, the original fasting-subsample weight (WTSAF2YR) was multiplied by 2/9.2 for each 2-year cycle. For NHANES 2017–March 2020, the pre-pandemic fasting-subsample weight (WTSAFPRP) was multiplied by 3.2/9.2. Masked variance strata and primary sampling units were incorporated into the survey design.

Serum CRP concentrations from 2005–2010 (LBXCRP) were converted from mg/dL to mg/L. Values below the detection limit were retained at the CDC-assigned fill value of 0.01 mg/dL, equivalent to 0.10 mg/L. For 2017–March 2020, hs-CRP concentrations (LBXHSCRP) were reported in mg/L. Values below the 0.15 mg/L detection limit were retained at the CDC-provided fill value of approximately 0.11 mg/L. Conversion to common units did not imply analytical equivalence between assay platforms.

**S2. Exposure standardization and quartile classification**

Exposure scales were established in the primary NHANES analytic sample and remained fixed throughout subsequent analyses. The survey-weighted standard deviations were 0.504973 for ln(calculated RC), 1.198933 for ln(cycle-specific CRP), and 1.403742 for ln(RCII).

Survey-weighted ln(RCII) quartile boundaries were 2.629728, 3.553060, and 4.481872. These boundaries and exposure-specific standard deviations were retained in restricted-sample sensitivity analyses. The same NHANES-derived standard deviations were applied to the outcome-enriched clinical sample.

**S3. Regression model specifications**

Model 1 was unadjusted. Model 2 included age, sex, race and ethnicity, educational attainment, marital status, and survey period. Model 3 additionally included family income-to-poverty ratio, current smoking, alcohol use, body mass index, diabetes, hypertension, and cardiovascular disease history.

Age, family income-to-poverty ratio, and body mass index were modeled continuously; remaining covariates were modeled categorically. When a sensitivity analysis excluded participants with a specific condition, the corresponding covariate was omitted if it became constant. Subgroup analyses likewise omitted the variable used to define the corresponding subgroup.

S4. Component contrasts and interaction analyses

Component-specific analyses included separate models for standardized ln(calculated RC), standardized ln(cycle-specific CRP), and standardized ln(RCII), followed by a mutually adjusted model containing both standardized components. A design-adjusted joint Wald test evaluated whether both component coefficients were zero.

The standardized coefficient contrast tested:

*H*_0_: β_ln(calculated RC)_ - β_ln(cycle-specific CRP)_ =0

A separate model using the unstandardized logarithmic components tested the equal-coefficient constraint implied by:

ln(RCII) = ln(calculated RC)+ ln(cycle-specific CRP)

Subgroup categories were sex, age 50–64 versus ≥65 years, and body mass index <25, 25–29.9, or ≥30 kg/m². Multiplicative interaction terms were assessed in the full analytic sample, and the three subgroup interaction tests were additionally evaluated using the Benjamini–Hochberg false-discovery-rate procedure.

Survey-period-specific estimates were derived as contrasts from a single pooled interaction model rather than from separately fitted period-specific models. Bone-status-specific estimates were similarly obtained from pooled models containing an exposure-by-bone-status interaction.

**S5. Outcome-enriched clinical sample component analyses**

Clinical component analyses used ordinary logistic regression adjusted for age, sex, and body mass index. Standardized ln(calculated RC), ln(hs-CRP), and ln(RCII) were scaled using the corresponding fixed NHANES survey-weighted standard deviations.

Separate and mutually adjusted component models were fitted. A joint Wald test assessed both component coefficients simultaneously, and a prespecified contrast compared their standardized coefficients. Because clinical participants were selected according to PHQ-9 screening status, analyses were interpreted in terms of exposure–outcome odds ratios rather than population prevalence or absolute risk.

**S6. Statistical software**

Analyses were conducted in R version 4.6.1 using the survey package, version 4.5. Variances were estimated using Taylor series linearization, with adjustment for strata containing a single primary sampling unit. All statistical tests were two-sided.

**Supplementary Table S1. Survey-weighted characteristics of adults aged ≥50 years with study-defined low bone mass according to depressive-symptom screening status.**

| **Characteristic** | **Overall**  **(*N* = 1,743)** | **PHQ-9 <10**  **(*N* = 1,604)** | **PHQ-9 ≥10**  **(*N* = 139)** | ***P* value** |
| --- | --- | --- | --- | --- |
| No. of participants, unweighted | 1,743 | 1,604 | 139 | — |
| Age, years | 64.7 (0.3) | 64.9 (0.3) | 61.6 (0.8) | <0.001 |
| Family income-to-poverty ratio | 3.20 (0.06) | 3.26 (0.06) | 2.12 (0.19) | <0.001 |
| Body mass index, kg/m² | 27.1 (0.2) | 27.1 (0.2) | 26.9 (0.6) | 0.836 |
| Minimum T-score | -1.87 (0.02) | -1.86 (0.03) | -1.97 (0.08) | 0.162 |
| Calculated remnant cholesterol, mg/dL | 20.0 [15.0, 29.0] | 20.0 [15.0, 28.0] | 24.0 [17.0, 33.0] | 0.026 |
| Cycle-specific C-reactive protein, mg/L | 1.60 [0.70, 3.63] | 1.50 [0.70, 3.55] | 2.80 [1.20, 6.40] | <0.001 |
| ln(RCII) | 3.56 (0.05) | 3.52 (0.06) | 4.18 (0.15) | <0.001 |
| Female sex | 1,116 (69.6%) | 1,020 (69.4%) | 96 (73.5%) | 0.440 |
| **Race/ethnicity** |  |  |  | 0.072 |
| Mexican American | 246 (4.4%) | 215 (4.1%) | 31 (8.6%) |  |
| Other Hispanic | 164 (4.0%) | 151 (4.0%) | 13 (3.6%) |  |
| Non-Hispanic White | 996 (80.3%) | 927 (81.0%) | 69 (69.8%) |  |
| Non-Hispanic Black | 199 (5.1%) | 182 (5.0%) | 17 (7.4%) |  |
| Other race/multiracial | 138 (6.2%) | 129 (5.9%) | 9 (10.6%) |  |
| **Educational attainment** |  |  |  | <0.001 |
| Above high school | 817 (56.6%) | 777 (58.2%) | 40 (31.7%) |  |
| High school or GED | 446 (27.7%) | 403 (27.0%) | 43 (39.1%) |  |
| Below high school | 480 (15.6%) | 424 (14.8%) | 56 (29.1%) |  |
| **Marital status** |  |  |  | 0.005 |
| Married or cohabiting | 1,014 (62.6%) | 959 (63.7%) | 55 (45.1%) |  |
| Never married | 84 (3.8%) | 72 (3.7%) | 12 (5.4%) |  |
| Widowed/divorced/separated | 645 (33.6%) | 573 (32.6%) | 72 (49.5%) |  |
| Current smoking | 253 (14.4%) | 211 (13.0%) | 42 (36.0%) | <0.001 |
| Alcohol use | 1,256 (77.3%) | 1,157 (77.2%) | 99 (78.7%) | 0.728 |
| Diabetes | 279 (11.1%) | 251 (10.8%) | 28 (15.0%) | 0.210 |
| Hypertension | 874 (45.0%) | 794 (44.3%) | 80 (56.3%) | 0.073 |
| Cardiovascular disease | 335 (16.4%) | 293 (15.6%) | 42 (27.8%) | 0.002 |

Note. Continuous variables are presented as survey-weighted mean (standard error), except calculated remnant cholesterol and cycle-specific CRP, which are presented as survey-weighted median [25th, 75th percentiles]. Categorical variables are presented as unweighted number (survey-weighted percentage). All estimates incorporated the pooled fasting-subsample weights, masked variance strata, and primary sampling units of the NHANES complex survey design. *P* values are two-sided and unadjusted. Mean-based continuous variables were compared using survey-weighted linear regression. Calculated remnant cholesterol and cycle-specific CRP were natural-log transformed for hypothesis testing; therefore, their *P* values test differences on the log scale rather than differences in weighted medians. Categorical variables were evaluated using Rao–Scott design-adjusted global tests. No variables included in the table were missing in the complete-case analytic sample. CRP, C-reactive protein; GED, General Educational Development; PHQ-9, Patient Health Questionnaire-9; RCII, remnant cholesterol–inflammation index; SE, standard error.

**Supplementary Table S2. Survey-weighted associations between ln(RCII) and continuous PHQ-9 scores.**

| **ln(RCII)** | **Model 1** | | **Model 2** | | **Model 3** | |
| --- | --- | --- | --- | --- | --- | --- |
|  | **β (95% CI)** | ***P value*** | **β (95% CI)** | ***P value*** | **β (95% CI)** | ***P value*** |
| Continuous, per weighted SD | 0.58 (0.34–0.82) | <0.001 | 0.55 (0.31–0.80) | <0.001 | 0.38 (0.13–0.63) | 0.004 |
| Quartile 1 | Reference | — | Reference | — | Reference | — |
| Quartile 2 vs 1 | −0.20 (−0.80–0.41) | 0.519 | −0.16 (−0.69–0.38) | 0.563 | −0.22 (−0.75–0.31) | 0.409 |
| Quartile 3 vs 1 | 0.42 (−0.20–1.04) | 0.182 | 0.41 (−0.14–0.95) | 0.144 | 0.12 (−0.45–0.69) | 0.666 |
| Quartile 4 vs 1 | 1.35 (0.71–2.00) | <0.001 | 1.27 (0.66–1.89) | <0.001 | 0.80 (0.17–1.44) | 0.015 |
| *P for trend* |  | <0.001 |  | <0.001 |  | 0.008 |

Note. Beta coefficients and 95% confidence intervals were estimated using survey-weighted linear regression with PHQ-9 total score as a continuous outcome. Model 1 was unadjusted. Model 2 adjusted for age, sex, race/ethnicity, educational attainment, marital status, and survey cycle. Model 3 additionally adjusted for family income-to-poverty ratio, current smoking, alcohol use, body mass index, diabetes, hypertension, and cardiovascular disease history. P for trend was calculated by assigning participants in each quartile the survey-weighted median ln(RCII) value for that quartile and modeling this value continuously. The weighted SD of ln(RCII) was 1.404; weighted quartile cut points were 2.630, 3.553, 4.482. CI, confidence interval; PHQ-9, Patient Health Questionnaire-9; RCII, remnant cholesterol–inflammation index; SD, standard deviation.

**Supplementary Table S3. Exploratory component-specific analysis in the outcome-enriched clinical sample.**

| **Model or test** | **Exposure or contrast** | **Effect estimate (95% CI)** | ***P* value** |
| --- | --- | --- | --- |
| Single-component model | ln(calculated RC), per 1 NHANES survey-weighted SD | OR 1.56 (1.01–2.40) | 0.044 |
| Single-component model | ln(hs-CRP), per 1 NHANES survey-weighted SD | OR 2.93 (1.77–4.86) | <0.001 |
| Mutually adjusted component model | ln(calculated RC), per 1 NHANES survey-weighted SD | OR 1.45 (0.92–2.28) | 0.113 |
| Mutually adjusted component model | ln(hs-CRP), per 1 NHANES survey-weighted SD | OR 2.86 (1.72–4.76) | <0.001 |
| Joint Wald test | H0: β[ln(calculated RC)] = β[ln(hs-CRP)] = 0 | χ²(2)=18.94 | <0.001 |
| Standardized coefficient contrast | β[standardized ln(calculated RC)] − β[standardized ln(hs-CRP)] | −0.682 (−1.370 to 0.006) | 0.052 |

Note. Models included all 150 participants (50 screening-positive events) and were adjusted for age, sex, and BMI. Exposures were standardized using fixed NHANES survey-weighted SDs: ln(calculated RC), 0.504973; ln(hs-CRP), 1.198933; and ln(RCII), 1.403742. Component results and the formal coefficient contrast are exploratory and should not be interpreted as biological contribution proportions.

**Supplementary Table S4. Sensitivity analyses of the association between ln(RCII) and depressive-symptom screening positivity.**

| **Analysis** | ***N*, unweighted** | **Screening-positive, *n*** | **OR per 1 fixed NHANES SD** | ***P* value** | **Q4 vs Q1 OR (95% CI)** | ***P* value** |
| --- | --- | --- | --- | --- | --- | --- |
| Primary analytic sample | 1,743 | 139 | 1.52 (1.15–2.00) | 0.004 | 3.00 (1.40–6.43) | 0.005 |
| Excluding CVD | 1,408 | 97 | 1.63 (1.19–2.22) | 0.003 | 3.80 (1.76–8.22) | 0.001 |
| Excluding diabetes | 1,464 | 111 | 1.49 (1.10–2.02) | 0.011 | 2.97 (1.27–6.95) | 0.013 |
| Excluding hypertension | 869 | 59 | 1.56 (1.12–2.16) | 0.009 | 2.71 (1.21–6.06) | 0.017 |
| Excluding CVD, diabetes, and hypertension | 723 | 43 | 1.41 (1.03–1.91) | 0.031 | 2.73 (1.39–5.37) | 0.004 |
| Restricting cycle-specific CRP to ≤10 mg/L | 1,605 | 120 | 1.56 (1.07–2.28) | 0.021 | 2.67 (1.16–6.13) | 0.021 |

Note. Odds ratios and 95% confidence intervals were estimated using survey-weighted logistic regression with the fully adjusted Model 3 specification. The survey-weighted standard deviation and quartile cut points of ln(RCII) were estimated in the primary NHANES sample and retained unchanged in all restricted-sample analyses. The fixed standard deviation was 1.403742, and the weighted quartile cut points were 2.629728, 3.553060, and 4.481872. When an exclusion criterion rendered a covariate constant, that covariate was omitted from the corresponding restricted-sample model. CI, confidence interval; CVD, cardiovascular disease; CRP, C-reactive protein; OR, odds ratio; RCII, remnant cholesterol–inflammation index; SD, standard deviation.

**Supplementary Table S5. Survey-period heterogeneity in the association between ln(RCII) and depressive-symptom screening positivity.**

| **Survey period** | **N, unweighted** | **Events, unweighted** | **Adjusted OR per 1 weighted SD (95% CI)** | ***P* value** |
| --- | --- | --- | --- | --- |
| 2005–2006 | 313 | 18 | 1.53 (0.91–2.58) | 0.105 |
| 2007–2008 | 429 | 39 | 1.27 (0.76–2.10) | 0.352 |
| 2009–2010 | 431 | 36 | 1.81 (1.24–2.64) | 0.003 |
| 2017–March 2020 | 570 | 46 | 1.60 (1.04–2.45) | 0.033 |

Note. Period-specific estimates were derived as linear contrasts from the pooled survey-weighted interaction model, not from separately fitted single-period models. The design-adjusted global interaction test was F(3, 48) = 0.52 (P = 0.674). Therefore, period-specific P values should not be interpreted as evidence that the association was present in some periods but absent in others. CI, confidence interval; OR, odds ratio; RCII, remnant cholesterol–inflammation index; SD, standard deviation.

**Supplementary Table S6. Effect modification by bone status in the association of ln(RCII) with depressive-symptom outcomes.**

**Panel A. Depressive-symptom screening positivity (PHQ-9 ≥10)**

| **Bone status / contrast** | **Model 1** | | **Model 2** | | **Model 3** | |
| --- | --- | --- | --- | --- | --- | --- |
|  | **OR (95% CI)** | ***P value*** | **OR (95% CI)** | ***P value*** | **OR (95% CI)** | ***P value*** |
| Normal bone | 1.60 (1.24–2.08) | <0.001 | 1.53 (1.16–2.00) | 0.003 | 1.33 (1.03–1.73) | 0.031 |
| Low bone mass | 1.62 (1.27–2.07) | <0.001 | 1.56 (1.20–2.01) | 0.001 | 1.37 (1.04–1.79) | 0.025 |
| Ratio of ORs | 1.01 (0.72–1.42) | 0.939 | 1.02 (0.72–1.45) | 0.914 | 1.02 (0.73–1.45) | 0.888 |

**Panel B. Continuous PHQ-9 score**

| **Bone status / contrast** | **Model 1** | | **Model 2** | | **Model 3** | |
| --- | --- | --- | --- | --- | --- | --- |
|  | **β (95% CI)** | ***P value*** | **β (95% CI)** | ***P value*** | **β (95% CI)** | ***P value*** |
| Normal bone | 0.60 (0.42–0.79) | <0.001 | 0.47 (0.28–0.65) | <0.001 | 0.25 (0.08–0.42) | 0.005 |
| Low bone mass | 0.58 (0.34–0.82) | <0.001 | 0.54 (0.31–0.78) | <0.001 | 0.31 (0.08–0.54) | 0.010 |
| Difference in β | −0.02 (−0.35–0.30) | 0.898 | 0.08 (−0.23–0.39) | 0.618 | 0.06 (−0.24–0.36) | 0.692 |

Note. Estimates were derived from survey-weighted regression models fitted in the full analysis sample and including an interaction term between ln(RCII) and low-bone-mass status. The normal-bone group included 1,999 participants (142 screening-positive events), and the low-bone-mass group included 1,743 participants (139 screening-positive events). ln(RCII) was modeled per 1 fixed survey-weighted standard deviation (1.403742). For Panel A, stratum-specific odds ratios and ratios of odds ratios were derived from the same survey-weighted logistic interaction model. For Panel B, stratum-specific β coefficients and between-stratum differences in β were derived from the same survey-weighted linear interaction model. The *P* values in the “Ratio of ORs” and “Difference in β” rows are *P* values for interaction. Model 1 was unadjusted. Model 2 adjusted for age, sex, race/ethnicity, educational attainment, marital status, and survey cycle. Model 3 additionally adjusted for family income-to-poverty ratio, smoking status, alcohol use, body mass index, diabetes, hypertension, and cardiovascular disease history. A significant within-stratum association does not by itself establish effect modification; effect modification was evaluated using the interaction *P* value. CI, confidence interval; OR, odds ratio; PHQ-9, Patient Health Questionnaire-9; RCII, remnant cholesterol–inflammation index.

**Supplementary Figure S1. Subgroup associations between ln(RCII) and depressive-symptom screening positivity.**

Odds ratios (ORs) and 95% confidence intervals (CIs) were estimated using survey-weighted logistic regression and are presented per 1 survey-weighted standard-deviation increase in ln(RCII). The overall estimate was derived from the fully adjusted model. Subgroup-specific models were adjusted for all covariates included in Model 3 except the variable used to define the corresponding subgroup. P values for interaction were obtained by testing the cross-product term between continuous ln(RCII) and each subgroup variable. Squares indicate subgroup-specific ORs, horizontal lines indicate 95% CIs, and the diamond indicates the overall estimate. The dashed vertical line denotes OR = 1. RCII, remnant cholesterol–inflammation index; OR, odds ratio; CI, confidence interval; BMI, body mass index.

**Supplementary Figure S2. Survey-weighted restricted cubic spline associations between ln(RCII) and depressive-symptom screening positivity across three adjustment models.**

**Note.** Restricted cubic spline models were fitted using survey-weighted logistic regression, with depressive-symptom screening positivity defined as a Patient Health Questionnaire-9 score ≥10. Knots were placed at the survey-weighted 10th, 50th, and 90th percentiles of ln(RCII), corresponding to 1.766, 3.553, and 5.472, respectively. The survey-weighted median of 3.553 was used as the reference. Red curves represent estimated odds ratios, and shaded areas represent 95% confidence intervals. The horizontal dashed line indicates an odds ratio of 1, and the vertical dotted line indicates the reference value. Model 1 was unadjusted. Model 2 was adjusted for age, sex, race and ethnicity, educational attainment, marital status, and survey period. Model 3 was additionally adjusted for family income-to-poverty ratio, current smoking, alcohol use, body mass index, diabetes, hypertension, and cardiovascular disease history. The overall test jointly evaluated all spline terms, whereas the nonlinearity test evaluated the nonlinear term while retaining the linear component. CI, confidence interval; OR, odds ratio; RCII, remnant cholesterol–inflammation index.

**Supplementary Table S7. Covariate dictionary for the NHANES Model 3 analysis**

| **Model covariate** | **Final analysis field** | **NHANES source variable(s)** | **Category definition / harmonization** | **Model coding and reference level** | **Refused, don't know, invalid, and missing handling** |
| --- | --- | --- | --- | --- | --- |
| Age | age_years (model: age) | RIDAGEYR | Age in completed years at screening. The primary analytic sample was restricted to age ≥50 years (observed range, 50-85). | Continuous; coefficient per 1-year increase. No categorical reference level. | Blank or nonnumeric values were set to missing. Missing age was excluded before the complete-case step; no missing age remained in N=1,743. |
| Sex | sex | RIAGENDR | 1=Male (n=627); 2=Female (n=1,116). | Categorical; Male was the reference level. The fitted model contained the indicator sexFemale. | Values other than 1 or 2 and blank values were set to missing; complete-case exclusion was applied. |
| Race/ethnicity | race_ethnicity (model: race) | RIDRETH1 | 1=Mexican American (n=246); 2=Other Hispanic (n=164); 3=non-Hispanic White (n=996); 4=non-Hispanic Black (n=199); 5=Other race, including multiracial (n=138). The same five-category RIDRETH1 recode was used across included releases. | Categorical; Mexican American was the reference level. | Blank or invalid values were set to missing; complete-case exclusion was applied. |
| Educational attainment | education | DMDEDUC2 | Codes 1 (<9th grade) and 2 (9th-11th grade, including 12th grade without a diploma) were grouped as Below high school (n=480); code 3 as High school/GED (n=446); codes 4 (some college/AA degree) and 5 (college graduate or above) as Above high school (n=817). | Categorical; Above high school was the reference level. | Codes 7 (refused), 9 (don't know), blank, and other invalid values were set to missing; complete-case exclusion was applied. |
| Marital status | marital_status (model: marital) | DMDMARTL (2005-2010); DMDMARTZ (2017-March 2020) | DMDMARTL: codes 1 (married) and 6 (living with partner) → Married/cohabiting; codes 2, 3, and 4 → Widowed/divorced/separated; code 5 → Never married. DMDMARTZ: code 1 → Married/cohabiting; code 2 → Widowed/divorced/separated; code 3 → Never married. Final counts: 1,014; 645; and 84, respectively. | Categorical; Married/cohabiting was the reference level. | DMDMARTL codes 77/99 and DMDMARTZ codes 77/99 (refused/don't know), blank, and invalid values were set to missing; complete-case exclusion was applied. |
| Family poverty-to-income ratio | poverty_income_ratio (model: pir) | INDFMPIR | Family income divided by the applicable federal poverty threshold; the public-use value is top-coded at 5.00 (observed range, 0.00-5.00). | Continuous; coefficient per 1-unit increase. No categorical reference level. | Blank or nonnumeric values were set to missing; complete-case exclusion was applied. No missing value remained in N=1,743. |
| Survey period | survey_cycle (model: cycle) | NHANES release/cycle identifier (SDDSRVYR-derived) | 2005-2006 (n=313), 2007-2008 (n=429), 2009-2010 (n=431), and the 2017-March 2020 pre-pandemic release (n=570). | Categorical; 2005-2006 was the reference level. | A valid included release was required. No missing survey-period value remained in N=1,743. |
| Current smoking | current_smoking_1yes (model: smoking) | SMQ020; SMQ040 | Yes (1; n=253): SMQ020=1 (≥100 cigarettes in life) and SMQ040=1 or 2 (now smokes every day or some days). No (0; n=1,490): SMQ020=2, or SMQ020=1 and SMQ040=3 (not at all). Thus former and never smokers were combined as noncurrent smokers. | Binary categorical; No (0) was the reference level. | Codes 7 (refused), 9 (don't know), blank, and logically unresolved combinations were set to missing; complete-case exclusion was applied. |
| Alcohol-use indicator | alcohol_use_1yes (model: drinking) | ALQ101 (2005-2010); ALQ111 (2017-March 2020) | 2005-2010: ALQ101=1 denoted ≥12 alcoholic drinks in any one year; ALQ101=2 denoted no. 2017-March 2020: ALQ111=1 denoted ever having ≥1 alcoholic drink (excluding small tastes/sips); ALQ111=2 denoted no. Final coding was Yes=1 (n=1,256) and No=0 (n=487). Because the NHANES question changed, this is a cycle-specific alcohol-exposure indicator rather than one identical behavioral threshold across all periods. | Binary categorical; No (0) was the reference level. | Codes 7 (refused), 9 (don't know), blank, and invalid values were set to missing; complete-case exclusion was applied. |
| Body mass index | BMI_kg_m2 (model: bmi) | BMXBMI | Measured body mass index in kg/m² (observed range, 15.25-54.40). | Continuous; coefficient per 1-kg/m² increase. No categorical reference level. | Blank, nonnumeric, or nonfinite values were set to missing; complete-case exclusion was applied. No missing value remained in N=1,743. |
| Diabetes | diabetes_1yes (model: diabetes) | DIQ010 | Self-reported physician diagnosis: DIQ010=1 (Yes; n=279) and DIQ010=2 (No; n=1,464). Row-level audit of the sample showed exact concordance between the final binary field and DIQ010. HbA1c was not part of the diabetes covariate used in the Model 3. | Binary categorical; No (0) was the reference level. | Borderline, refused, don't know, blank, and other values outside the accepted yes/no codes were treated as missing; complete-case exclusion was applied. |
| Hypertension | hypertension_1yes (model: hypertension) | BPQ020 | Self-reported physician diagnosis: BPQ020=1 (Yes; n=874) and BPQ020=2 (No; n=869). Row-level audit of the sample showed exact concordance between the final binary field and BPQ020. Antihypertensive medication use and measured blood-pressure thresholds were not part of the hypertension covariate used in the Model 3. | Binary categorical; No (0) was the reference level. | Codes 7 (refused), 9 (don't know), blank, and invalid values were set to missing; complete-case exclusion was applied. |
| Cardiovascular disease history | cardiovascular_disease_1yes (model: cvd) | MCQ160B, MCQ160C, MCQ160D, MCQ160E, MCQ160F | Composite of self-reported congestive heart failure, coronary heart disease, angina/angina pectoris, heart attack, or stroke. Yes (1; n=335) if any component was coded 1; No (0; n=1,408) only when all five components were coded 2. | Binary categorical; No (0) was the reference level. | For each item, codes 7 (refused), 9 (don't know), blank, and invalid values were set to missing. A participant without any positive component but with one or more missing components was coded missing for the composite; complete-case exclusion was applied. |

**Notes.** All covariates were prespecified in Model 3. Age, poverty-to-income ratio, and BMI were entered continuously; all other covariates were categorical. The actual reference levels were verified from the fitted-model coefficient names. The primary analysis used complete cases for the exposure, outcome, survey-design variables, and all Model 3 covariates; no imputation was performed. Consequently, the analytic sample (N = 1,743) has zero missing values for every covariate listed above.

AA, associate degree; BMI, body mass index; GED, General Educational Development; NHANES, National Health and Nutrition Examination Survey.
